# Subtyping of *Clostridium difficile* PCR ribotypes 591, 106 and 002, the dominant strain types circulating in Medellin, Colombia

**DOI:** 10.1371/journal.pone.0195694

**Published:** 2018-04-12

**Authors:** Clara Lina Salazar, Catalina Reyes, Astrid Vanessa Cienfuegos-Gallet, Emma Best, Santiago Atehortua, Patricia Sierra, Margarita M. Correa, Warren N. Fawley, Daniel Paredes-Sabja, Mark Wilcox, Angel Gonzalez

**Affiliations:** 1 Research Group in Anaerobic Bacteria (GIBA), School of Microbiology, Universidad de Antioquia, Medellín, Colombia; 2 Basic and Applied Microbiology Research Group (MICROBA), School of Microbiology, Universidad de Antioquia, Medellín, Colombia; 3 Departament of Microbiology, Leeds Teaching Hospitals NHS Trust, Leeds, United Kingdom; 4 San Vicente Fundación University Hospital, Medellín, Colombia; 5 Clínica León XIII, IPS Universitaria, Universidad de Antioquia, Medellín, Colombia; 6 Molecular Microbiology Group, School of Microbiology, Universidad de Antioquia, Medellín, Colombia; 7 Microbiota-Host Interactions and Clostridia Research Group, Departamento de Ciencias Biológicas, at Universidad Andres Bello, Santiago, Chile; Defense Threat Reduction Agency, UNITED STATES

## Abstract

We aimed to achieve a higher typing resolution within the three dominant *Clostridium difficile* ribotypes (591,106 and 002) circulating in Colombia. A total of 50 *C*. *difficile* isolates we had previously typed by PCR-ribotyping, representing the major three ribotypes circulating in Colombia, were analyzed. Twenty-seven isolates of ribotype 591, 12 of ribotype 106 and 11 of ribotype 002 were subtyped by multiple locus variable-number tandem-repeat analysis (MLVA). The presence of the PaLoc genes (*tcd*A/*tcd*B), toxin production in culture and antimicrobial susceptibility were also determined. From the total *C*. *difficile* ribotypes analyzed, 20 isolates (74%) of ribotype 591, nine (75%) of ribotype 106 and five (45.5%) of ribotype 002 were recovered from patients with *Clostridium difficile* infection (CDI). MLVA allowed us to recognize four and two different clonal complexes for ribotypes 591 and 002, respectively, having a summed tandem-repeat difference (STRD) <2, whereas none of the ribotype 106 isolates were grouped in a cluster or clonal complex having a STRD >10. Six ribotype 591 and three ribotype 002 isolates belonging to a defined clonal complex were isolated on the same week in two different hospitals. All ribotypes harbored either *tcd*A^+^/*tcd*B^+^ or *tcd*A^-^/*tcd*B^+^ PaLoc genes. Moreover, 94% of the isolates were positive for toxin in culture. All isolates were susceptible to vancomycin and metronidazole, while 75% to 100% of the isolates were resistant to clindamycin, and less than 14.8% of ribotype 591 isolates were resistant to moxifloxacina. No significant differences were found among ribotypes with respect to demographic and clinical patients’ data; however, our results demonstrated a high molecular heterogeneity of *C*. *difficile* strains circulating in Colombia.

## Introduction

*Clostridium difficile* infection (CDI) is considered the major cause of infectious diarrhea in healthcare environments [1]. This infection is associated with the development of mild diarrhea to pseudomembranous colitis and toxic megacolon [2]. The incidence and severity of this bacterial disease have increased worldwide in the last years due to the spreading of the hypervirulent and epidemic strain known as PCR ribotype 027 [3,4]. Nonetheless, several studies have demonstrated the change on the epidemiology of *C*. *difficile* in Europe and North America; thus, ribotypes 014/020, 014, 001, 001/072 and 078 are currently more prevalent than ribotype 027 [1,5].

In Latin America, the presence of the virulent strain 027 has also been reported in Mexico, Costa Rica, Panama and Chile [6–9]. Recently in Colombia, *C*. *difficile* ribotype 027 was documented in three CDI patients using the commercial assay Xpert® *C*. *difficile* [10]. Furthermore, a previous study applying the PCR-ribotyping technique to 143 isolates obtained at three high-complexity hospitals of Medellín, Colombia demonstrated the presence of 37 different ribotypes the most frequent ones being 591, 106 and 002. Notably, only one isolate corresponded to ribotype 027 and four new ribotypes (795, 795, 804 and 805) were reported [11].

PCR-ribotyping which amplifies the polymorphic sequences in the 16S-23S intergenic spacer regions (ISR), is the most frequently employed molecular method for the global analysis of related *C*. *difficile* strains, including the epidemic clone; it is also considered the gold standard method for *C*. *difficile* typing [12–14]. However, this technique has lower discriminatory power, and by itself, is not appropriate to investigate cases of cross infection or to determine the source of epidemic outbreaks; thus, it is of limited utility in epidemiological studies [15,16]. Other methods used for typing *C*. *difficile* include pulsed-field gel electrophoresis (PFGE), which has low discriminatory power and restriction endonuclease analysis (REA), which shows a high discriminatory power, but they can sometimes be difficult to interpret a times [16]. Although multilocus variable-number tandem-repeat analysis (MLVA) is not as discriminatory as whole genome sequencing, this subtyping technique is useful for tracking bacterial transmission at a local level, especially in the case of *C*. *difficile* infections [17–19]. Therefore, in this study we evaluated the epidemiological significance of the clustering obtained by MLVA subtyping of the three major *C*. *difficile* Colombian ribotypes (591, 106 and 002).

## Material and methods

### Study design

This study was conducted between January 2013 and January of 2015 as a part of a cross-sectional study performed in three tertiary care hospitals (denoted by A, B and C) in Medellín, Colombia. *C*. *difficile* from patients with suspicion of CDI were isolated by culture and characterized by PCR ribotyping as previously described [20].

### Demographic and clinical data

A CDI case was defined if a patient had symptoms that included diarrhea and a stool positive for *C*. *difficile* A/B toxins [11,21]. A review of each patient’s medical record was performed in order to obtain and collect demographic (age, gender) and clinical data. Data from patients with other ribotypes were used to compare with equivalent data for patients with ribotypes 591, 106 and 002.

### Ethics statement

The Human Research Ethics Committee of Universidad de Antioquia (Comité de Bioética, Sede de Investigación Universitaria, CBEIH- SIU, approval number 12-35-458) approved this work. An informed consent was obtained in writing from all patients involved in this study. All patients’ data were anonymized and only available to the research group.

### *Clostridium difficile* strains

A total of 50 *C*. *difficile* strains that had previously been typed by PCR ribotyping were included in this study. All the isolates representing the major three ribotypes circulating in Colombia, as reported in a previous study [11], were selected. The major ribotypes were 591 (n = 27), 106 (n = 12) and 002 (n = 11). *C*. *difficile* was grown in brain-heart infusion (BHI) broth (Becton Dickinson, Franklin Lakes, NJ, USA), and its DNA was extracted using a DNA isolation kit (MoBio Laboratories Inc., Carlsbad, CA), following the manufacturers’ instructions.

### Molecular and phenotypic characterization of *C*. *difficile* strains

All *C*. *difficile* ribotypes (591, 106 and 002) analyzed in this study were further investigated by the presence of the toxin genes *tcd*A and *tcd*B using standardized methods [21]. The *tcd*A (toxin A) and *tcd*B (toxin B) genes were determined using the NK2/NK3 and NK104/NK105 primers, respectively, to amplify a 375-bp fragment for *tcd*A and a 203-bp fragment for *tcdB* [22]. Toxin production by *C*. *difficile* in culture was determined using a standardized cytotoxic assay [23]. Antimicrobial susceptibility to metronidazole, vancomycin, clindamycin and moxifloxacin was determined following guidelines by the Clinical and Laboratory Standards Institute (CLSI; Document M100-S23) [24]. *Bacillus fragilis* ATCC 25285 and *C*. *difficile* ATCC 500057 were used as resistant and susceptible control strains, respectively.

### Multiple-Locus Variable Tandem Repeats analysis (MLVA)

MLVA was performed for the identification of clonally related isolates within each ribotype. Seven regions within the *C*. *difficile* genome with tandem repeat loci were designated and amplified individually by PCR with specific primers A6, B7, C6, E7, F3, G8, and H9 as previously described [17–19,21], and using a single protocol [25]. Each forward primer, for all loci, was labeled with fluorescent dye at the 5’ end with either 6 carboxyfluorescein (FAM), hexachlorofluorescein (HEX), 2’-chloro-7’-phenyl-1,4-dichloro-6-carboxyfluorescein (VIC), or 2’-chloro-5’-fluoro-7’,8’-fused phenyl-1, 4-dichloro-6 carboxyfluorescein (NED) [25,26]. The PCR amplification conditions were adjusted for PCR ribotype 078 to improve amplification, because locus A6 is usually not present in isolates of this ribotype [26]. PCR products were analyzed using an ABI-PRIMS 3130xl automated sequencer and fragment analysis system (Life Technologies Ltd, Paisley, UK) with GeneScan 600 LIZ (Applied Biosystems, Life Technologies, Grand Island, NY) as an internal marker. Fragment sizes were determined with the GeneMapper v.4.0 software (Applied Biosystems). Repeat numbers were analyzed using Bio-Numerics software (Applied Maths, Kortrijk, Belgium). The summed absolute distance between two MLVA-typed isolates is the summed tandem-repeat difference (STRD) at all seven variable numbers of tandem-repeat (VNTR) loci.

To identify clustering among *C*. *difficile* isolates, we determined the number of differences in tandem repeats increases; thus, isolates differing by 0, 1, or 2 tandem repeats (STRD ≤2) were considered clonal complexes or indistinguishable, while those isolates that differed by up to 10 tandem repeats (STRD ≤ 10) were considered as genetically related [19,25]. The epidemiological significance of the clustering among CDI cases was further evaluated by adding the variables time and space in the analysis.

### Statistical analysis

Statistical analyses were performed in Stata v.13.0 (StataCorp LP, College Station, TX) and R software (version 3.3.3, Robert Gentleman and Ross Ihaka, Auckland, New Zealand). Data were described using absolute and relative frequencies for categorical variables and median and interquartile range for quantitative variables with non-normal distribution. Comparisons of categorical variables among groups of patients infected by each ribotype were done using chi-square or Fisher’s exact tests. Quantitative variables comparisons were done using ANOVA or Kruskal-Wallis tests, depending on the homoscedasticity assumption.

To explore the relationship among categories of qualitative variables related to clinical characteristics at the time of CDI suspicion, a multiple correspondence analysis (MCA) was performed. The following variables were included: fever ≥ 38.5 ^o^C, ileus, unconsciousness, abdominal distention, multiorgan failure, other infections and death. Quantitative variables were categorized to be included in the analysis. White blood cells (WBC) count was categorized according to the guidelines of the American College of Gastroenterology for *C*. *difficile* infection (non-severe: <15,000 cells/mm^3^, severe: 14,999–34,999 cells/mm^3,^ complicated disease: >35,000 cells/mm^3^) (20); C-Protein Reactive levels were categorized as low risk (<9.9 mg/L) and high risk levels (>10 mg /L).

## Results

### Epidemiological and demographic data of patients carrying the dominant *C*. *difficile* ribotypes 591, 106 and 002

Demographic and clinical data of patients with suspicion of CDI and carrying the ribotypes 591, 106 and 002 were compared with data from patients with suspicion of CDI and carrying other ribotypes (a total of 34 different ribotypes were included in this group) (Table 1). No significant differences were found with regard to age, gender, hospital service [internal medicine, surgery, hematology, intensive care unit (ICU), special care unit (SCU), orthopedic or nephrology], risk factors (older than 65 years, abdominal surgery, endoscopy, nasogastric tube, steroids usage, gastric acid suppressants, proton pump inhibitors, dialysis or stay in ICU before symptoms), laboratory data, clinical signs and symptoms (fever, abdominal distention, ileus, hypotension, loss of consciousness, multiorgan failure and colitis), discharge, other infections or previous usage of antibiotics, with the exception of macrolides usage, which was more frequently significant in those patients carrying ribotype 591 (Table 1).

**Table 1:** Demographic and clinical characteristics of patients carrying the 591, 106, 002 and other ribotypes.

	Ribotype 591	Ribotype 106	Ribotype 002	Other ribotypes	*P* value
	n (%)	n (%)	n (%)	n (%)	
	Total = 27	Total = 12	Total = 11	Total = 91	
**Male**	15 (55.6)	4 (33.3)	5 (45.5)	49 (53.8)	0.548^†^
**Female**	12 (44.4)	8 (66.7)	6 (54.5)	42 (46.2)	
**Age**					
Median	65	66	65	61	0.614^†^
IQR	49–81	23–79	47–76	45–75	
**Hospital service**					
Internal medicine	18 (66.7)	8 (66.7)	8 (72.7)	64 (70.3)	0.972^†^
Surgery	5 (18.5)	2 (16.7)	0	18 (19.8)	0.510^#^
Hematology	2 (7.4)	1 (8.3)	0	3 (3.3)	0.482^#^
ICU	4 (14.8)	2 (16.7)	2 (18.2)	13 (14.3)	0.930^#^
SCU	2 (7.4)	0	1 (9.1)	3 (3.3)	0.377^#^
Orthopedic	5 (18.5)	2 (16.7)	1 (9.1)	9 (9.9)	0.579^#^
Nephrology	2 (7.4)	1 (8.3)	1 (9.1)	9 (9.9)	1.000^#^
**Risk factors**					
Older 65 years	13 (48.1)	6 (50.0)	5 (45.5)	39 (42.9)	0.942^†^
Abdominal surgery	5 (18.5)	1 (8.3)	1 (9.1)	19 (20.9)	0.756^#^
Previous endoscopy	4 (14.8)	2 (16.7)	0	23 (25.3)	0.213^#^
Nasogastric tube	6 (22.2)	2 (16.7)	1 (9.1)	23 (25.3)	0.776^#^
Steroids	8 (29.6)	4 (33.3)	3 (27.3)	24 (26.4)	0.950^#^
Gastric acid suppressants	8 (29.6)	1 (8.3)	3 (27.3)	28 (30.8)	0.467^#^
Proton pump inhibitors	24 (88.9)	11 (91.7)	9 (81.8)	69 (75.8)	0.336^†^
Dialysis	7 (25.9)	3 (25.0)	2 (18.2)	12 (13.2)	0.313^#^
Stay ICU before symptoms	11 (40.7)	4 (33.3)	1 (9.1)	21 (23.1)	0.157^#^
**Laboratory Data**					
White blood cells					
Median	11700	11015	11330	8770	0.008^†^
IQR	6510–16800	9100–14425	9510–20260	6900–17600	
WBC severity scoring					
WBC < 15.000 cell/mm3*	19 (70.4)	10 (83.3)	8 (72.7)	63 (69.2)	
WBC 15.000–34.999 cells/mm3*	6 (22.2)	1 (8.3)	1 (9.1)	27 (29.7)	0.047^†^
WBC ≥ 35.000 cell/mm3*	2 (7.4)	1 (8.3)	2 (18.9)	1 (1.1)	
C-Reactive protein					
Median	7.86	8.35	9.96	9.14	0.036^†^
IQR	3.41–12.8	2.13–10.7	4.06–18.3	3.06–18.0	0.008^†^
**Clinical signs and symptoms**					
Fever	6 (22.2)	3 (25)	1 (9.1)	14 (15.4)	0.616^#^
Abdominal distention	5 (18.5)	3 (25)	2 (18.2)	26 (28.6)	0.603^#^
Ileus	4 (14.8)	0	0	2 (2.2)	0.707^#^
Hypotension	3 (11.1)	1 (8.3)	1 (9.1)	9 (9.9)	0.954^#^
Loss of consciousness	3 (11.1)	0	0	2 (2.2)	0.157^#^
Multiorgan failure	1 (3.7)	0	0	4 (4.4)	0.365^#^
Colitis by tomography	2 (7.4)	0	0	4 (4.4)	0.697^#^
Discharge**					
Death	5 (18.5)	2 (16.7)	1 (9.1)	17 (18.7)	0.856^#^
Improve symptoms	21 (77.8)	10 (83.3)	10 (90.9)	73 (80.2)	
Other infections***	14 (51.9)	6 (50.0)	5 (45.4)	54 (59.3)	0.736^†^
**Previous usage of antibiotics**					
Carbapenems	13 (48.1)	4 (33.3)	2 (18.2)	29 (31.9)	0.309^#^
Cephalosporins					
3rd generation	4 (14.8)	1 (8.3)	2 (18.2)	12 (13.2)	0.912^†^
4th generation	2 (7.4)	2 (16.7)	1 (9.1)	10 (10.9)	0.840^#^
Macrolides	7 (25.9)	0	1 (9.1)	5 (5.5)	0.014^#^
Metronidazole	3 (11.1)	2 (16.7)	2 (18.2)	11 (12.1)	0.820^#^

CDI, *Clostridium difficile* infection; ICU, intensive care unit; SCU, special care unit.

*According to severity scoring system of Guidelines for diagnosis, treatment and prevention of *Clostridium difficile* infections.

**One patient carrying the ribotype 591 was transferred to a different hospital

***Primary bacteremia, urinary tract infection, pneumonia.

^#^ Fisher’s exact test

^†^ Chi-square’s test

Of 50 patients with suspicion of CDI, 27 (54%) carried ribotype 591, 12 (24%) ribotype 106 and 11 (22%) ribotype 002. After classification of patients as CDI or non-CDI according to the free *C*. *difficile* A/B toxin in stool samples, it was found that 20 (74%) isolates of ribotype 591, 9 (75%) of ribotype 106 and 5 (45.5%) of ribotype 002 were recovered from CDI patients. Regarding the ribotype 591, 13 (48.1%) isolates were from hospital A, 12 (44.5%) from hospital B, and two (7.4%) from hospital C; 50% of the isolates ribotype 106 were from hospital A, and the other 50% from hospital B; eight (72.7%) isolates corresponding to ribotype 002 were from hospital A, 2 (18.2%) from hospital B and only one (9.1%) from hospital C.

An epidemic curve for the distribution of all ribotypes during the different periods of time in the three hospitals revealed no trend in the number of cases diagnosed per month.

**Fig 1:**
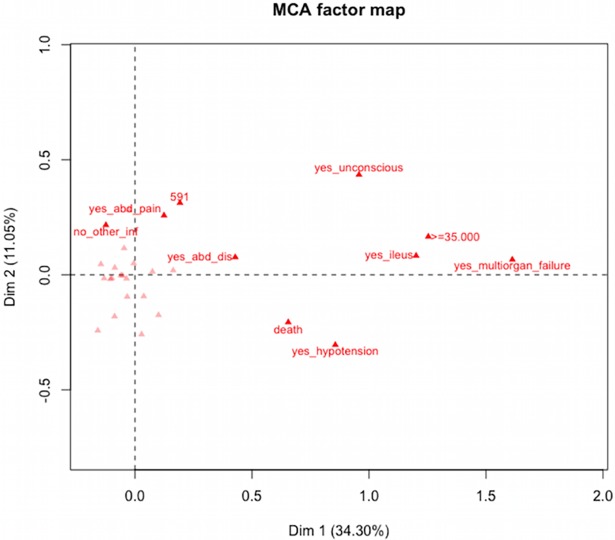
Multiple correspondence analyses. Variable categories contributing to dimension 1 (x-axis) and dimension 2 (y axis); variables with major contribution are close to the cero line in the chart. Ten variables contributing to both dimensions are shown.

The MCA showed that the first two dimensions explained 45.6% of total variation. The variables with higher significant contribution to both dimensions were multiorgan failure, followed by death, hypotension, WBC≥35,000 cells/mm^3^, ileus, abdominal distention and unconsciousness (Fig 1). Of note, ribotype 591 was related to abdominal pain and abdominal distention.

### Molecular and phenotypical characteristics of *C*. *difficile* ribotypes 591, 106 and 002

**Fig 2:**
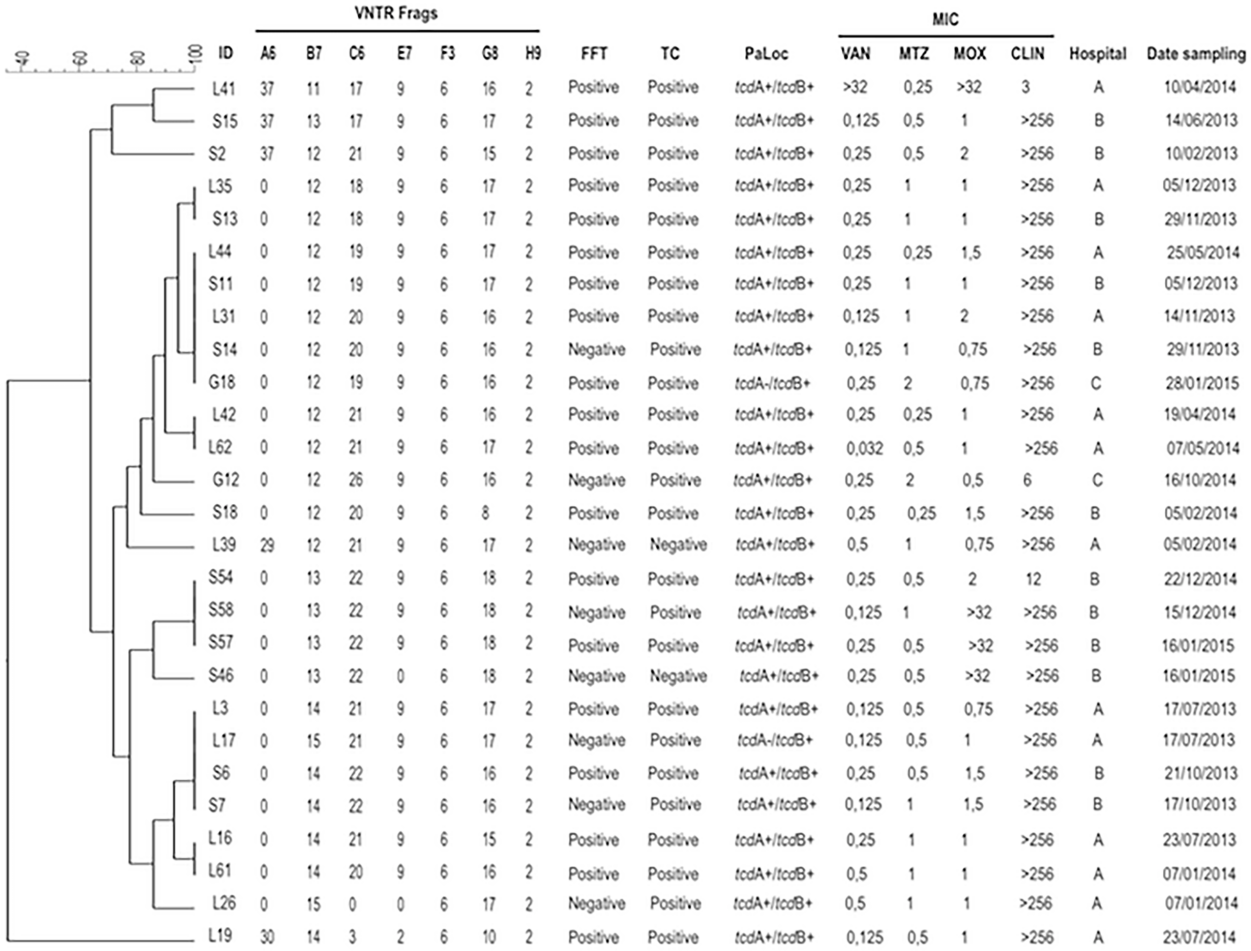
Analysis of ribotype 591 by MLVA. MLVA was applied to 27 isolates belonging to the ribotype 591. Dendrogram showing similarities of banding patterns generated with high-resolution capillary gel-based electrophoresis (CE)-PCR. MLVA clusters are identified by minimum-spanning tree: one clonal complex is defined by a MLVA type with less than a two-locus difference. In addition, free fecal toxin A/B (FFT), toxin in cell culture (TC), presence of PaLoc genes (*tcd*A/*tcd*B), minimal inhibitory concentrations (MIC) for vancomycin (VAN), metronidazole (MTZ), moxifloxacin (MOX) and clindamycin (CLIN) are shown for each isolate with the respective breakpoints. VNTR, variable-number tandem repeat.

**Fig 3:**
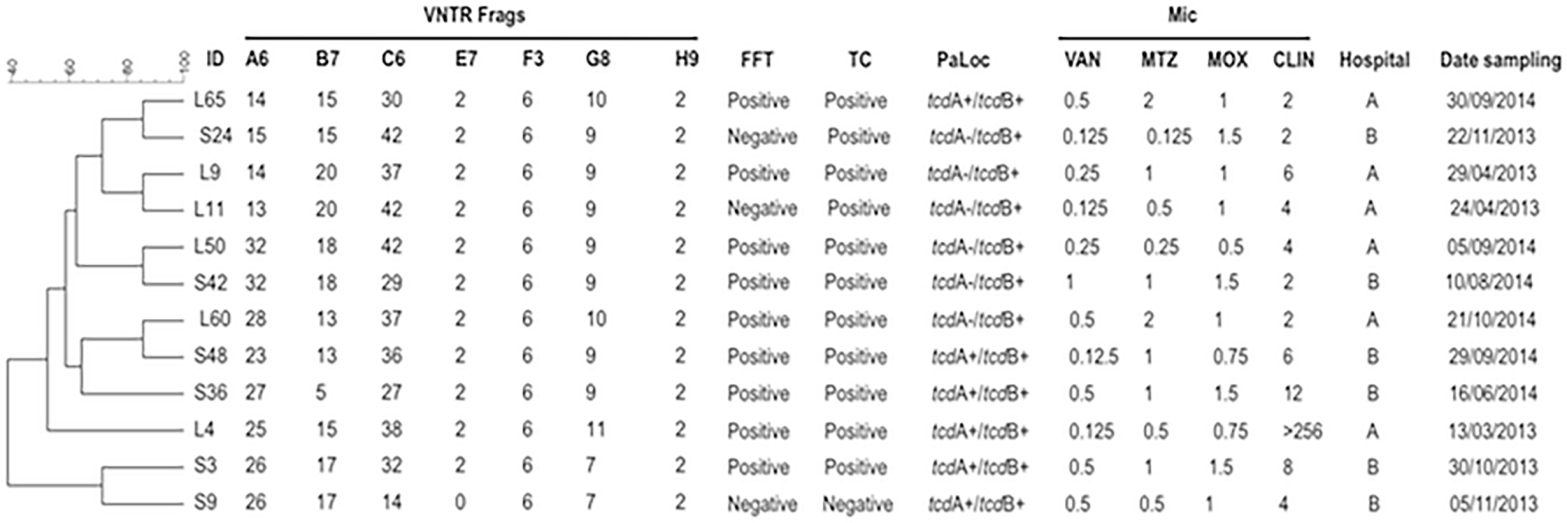
Analysis of ribotype 106 by MLVA. MLVA was applied to 12 isolates belonging to the ribotype 106. Dendrogram showing similarities of banding patterns generated with high-resolution capillary gel-based electrophoresis (CE)-PCR. MLVA clusters are identified by minimum-spanning tree: one clonal complex is defined by MLVA type with less than a two-locus difference. In addition, free fecal toxin A/B (FFT), toxin in cell culture (TC), presence of PaLoc genes (*tcd*A/*tcd*B), minimal inhibitory concentrations (MIC) for vancomycin (VAN), metronidazole (MTZ), moxifloxacin (MOX) and clindamycin (CLIN) are shown for each isolate with the respective breakpoints. VNTR, variable-number tandem repeat.

**Fig 4:**
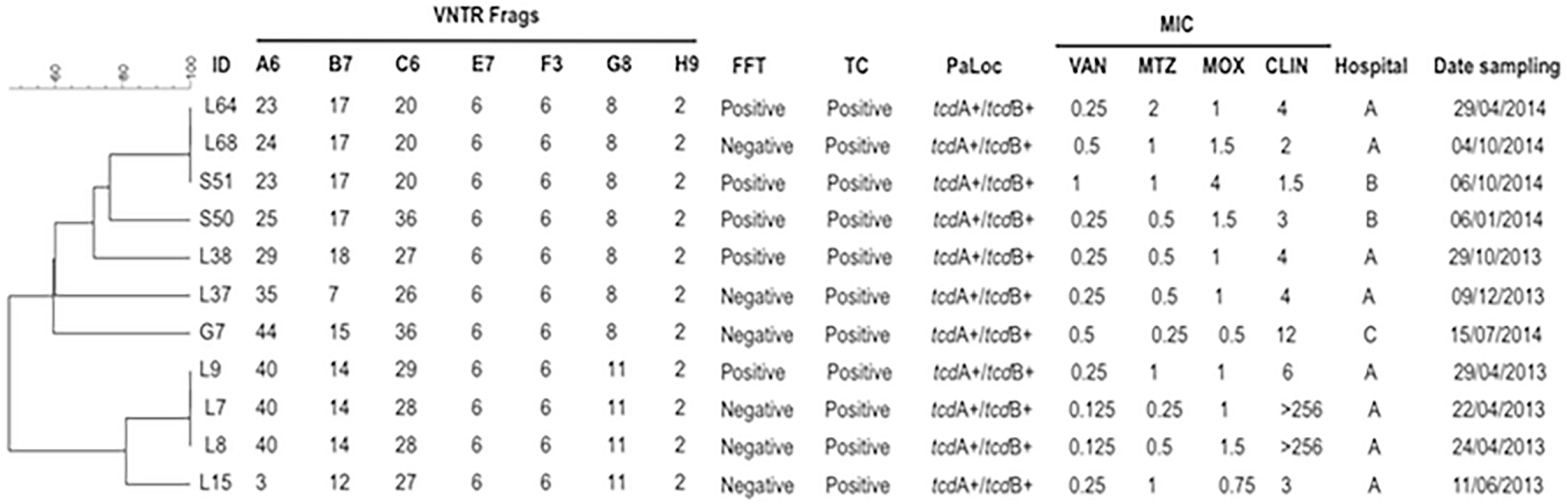
Analysis of ribotype 002 by MLVA. MLVA was applied to 11 isolates belonging to the ribotype 002. Dendrogram showing similarities of banding patterns generated with high-resolution capillary gel-based electrophoresis (CE)-PCR. MLVA clusters are identified by minimum-spanning tree: one clonal complex is defined by MLVA type with less than a two-locus difference. In addition, free fecal toxin A/B (FFT), toxin in cell culture (TC), presence of PaLoc genes (*tcd*A/*tcd*B), minimal inhibitory concentrations (MIC) for vancomycin (VAN), metronidazole (MTZ), moxifloxacin (MOX) and clindamycin (CLIN) are shown for each isolate with the respective breakpoints. VNTR, variable-number tandem repeat.

From the 27 isolates belonging to ribotype 591, 25 (92.6%) were positive for both PaLoc genes (*tcd*A^+^/*tcd*B^+^) and the remaining 2 (7.4%) were positive only for the gene coding for toxin B (*tcd*A^-^/*tcd*B^+^); of note, 92.6% of these isolates were positive for toxin production in cell culture (Fig 2). From the 12 isolates belonging to ribotype 106, 6 (50%) harbored the *tcd*A^+^/*tcd*B^+^ genes, the remaining 6 (53.8%) isolates were *tcd*A^-^/*tcd*B^+^, and 11 (84.6%) isolates were positive for toxin in cell culture (Fig 3). In connection with the ribotype 002, all isolates harbored the PaLoc genes and were positive for toxin in cell culture (Fig 4).

Regarding the susceptibility of *C*. *difficile* to antibiotics, all isolates corresponding to 591, 106 and 002 ribotypes were susceptible to vancomycin and metronidazole, 75% to 100% were resistant to clindamycin, and less than 15% of ribotypes 591 and 106 were resistant to moxifloxacina (Table 2). The breakpoints of MICs are shown for each isolate (Figs 2, 3 and 4).

**Table 2:** Antibiotic resistance profile of C. difficile strains of ribotypes 591, 106 and 002.

	*Clostridium difficile* ribotype		
	591	106	002
	n = 27 (%R)	n = 12 (%R)	n = 11 (%R)
**Antibiotic**			
Metronidazole	0	0	0
Vancomycin	0	0	0
Clindamycin	27 (100	9 (75)	9 (81.8)
Moxifloxacin	4 (14.8)	0	0

R: resistance

### MLVA subtyping

MLVA was performed on the *C*. *difficile* strains from the three predominant ribotypes. In the present study, a total of 4 clonal complexes were identified for ribotype 591 (Fig 2) and two clonal complexes for ribotype 002 (Fig 4) as defined by a STRD ≤ 2. Of interest, six isolates coming from hospital B and belonging to ribotype 591 and a specific clonal complex with a STRD ≤ 2 were distributed as follows: isolates S6 and S7 were isolated on an interval of five days on October of 2013, isolates S11 and S14 were isolated on an interval of seven days between November and December of 2013, and isolates S54 and S58 were isolated on an interval of eight days on December of 2014 (Fig 2). In a similar fashion, three isolates belonging to ribotype 002 (isolates L7, L8 and L9) with a STRD ≤ 2 and coming from hospital A were isolated on an interval of eight days (Fig 4). Importantly, all isolates from the same clonal complex were recovered from inpatients in different wards. For ribotype 106, none of the isolates belonged to a cluster or clonal complex with a STRD > 10 (Fig 3).

Of note, in 22 (81.5%) of the isolates ribotype 591 locus A6 did not amplify; in addition, locus C6 and E7 did not amplify in one isolate (Fig 2). A great diversity in the number of repetitions was noted especially for locus A6, B7 and C6, whereas locus E7, F3, G8 and H9 were the most constant and common among all three ribotypes analyzed (Figs 2, 3 and 4).

## Discussion

We previously reported that *C*. *difficile* ribotypes 591, 106 and 002 are the dominant strains circulating in Colombia [11]. Although PCR-ribotyping is considered the gold standard method for *C*. *difficile* typing and is the most employed for global analysis of related strains [12–14], this technique has a lower discriminatory power, and alone is insufficient to investigate cases of cross infection or epidemic outbreaks [15,16]. Subsequently, the MLVA technique was used since it has been widely used to subtype *C*. *difficile* strains [17,19,27]. Although whole genome sequencing has the higher discriminatory power than MLVA, Eyre et al. suggested that MLVA provides similar discrimination power, and the effective implementing of WGS has several barriers that need to be addressed [26]. MLVA was performed on 50 isolates of *C*. *difficile* belonging to the ribotypes 591, 106 and 002. Amplification was obtained for the loci A6, B7, C6, E7, F3, G8 and H9 as previously described. Of note, 81.5% of isolates belonging to ribotype 591 did not amplify the locus A6. This finding is in line with a previous study in which 33 strains of PCR ribotypes 023, 017, 078 and 126 did no amplify the locus A6 [28]; moreover, absence of amplification has been already reported, and it was explained by the absence of this locus in ribotype 078, as evidenced by sequence analysis [19,29,30]. Although in the present study no sequence analysis was performed, absence of this locus should not be ruled out. Additionally, we observed a great diversity in the number of repetitions in the locus A6, B7 and C6, results that are in accordance with other studies [15,28]. Thus, the diversity in the number of repetitions observed may be due to an intraspecimen variation within the host as a consequence of a rapid evolution of the microorganism.

MLVA results indicated that not all 27 isolates belonging to the ribotype 591 were closely related at the clonal level, since they showed different STRDs values; only four different clonal complexes with a STRD ≤ 2 were observed for this major ribotype. Regarding the ribotype 002, only two different clonal complexes with a STRD ≤ 2 were observed. None of the isolates belonging to ribotype 106 showed a cluster or clonal complex distribution even with a STRD < 10. Interestingly, six ribotype 591 isolates were shown to belong to three clonal complexes with a STRD ≤ 2, they came from hospital B and were isolated from six patients in three different time intervals; similarly, three isolates with the same ribotype 002 with a STRD < 2 and coming from hospital A were isolated from three patients over an interval of 8 days; however, all isolates were recovered from inpatients in different wards. Thus, contaminations of hospital environments due to persistence of spores or bacterial spread through asymptomatic carriers have been suggested as important risk factors for healthcare-associated infections [31]. Although in the present study we were not able to confirm the presence of *C*. *difficile* spores in the environment or transmission from health personnel to patients or vice versa, our findings clearly suggest a possible intra-hospital transmission within hospitals A and B due to of ribotypes 002 and 591.

Interestingly, our results clearly indicate a difference in diversity and epidemiology of *C*. *difficile* when compared with those studies carried out in Europe and United States, where the dominant *C*. *difficile* circulating strains were ribotypes 027, 001/072, 014/002 and 176 [1,32,33]. Regarding the demographic and clinical data of patients carrying ribotypes 591, 106 and 002, no significant differences were observed in any of the variables analyzed when these dominant ribotypes were compared with the others detected, and when comparing between CDI and non-CDI patients. Of note, the exploratory results of the MCA indicated that isolates of ribotype 591 were related to important clinical variables, and associated with presence of abdominal pain and abdominal distention. Nonetheless, further studies should be addressed to confirm this relation given the small number of samples analyzed.

We observed that some stool samples, in which toxigenic strains were isolated, are negative for *C*. *difficile* toxins; this could be a result of sensitivity to the diagnostic test (EIA) used. Due to variations in the sensitivity and specificity of the various diagnostic tests for CDI, the European Society of Clinical Microbiology and Infectious Diseases (ESCMID) recommends the use of a two-step algorithm to optimize diagnosis [34]. The use of one single EIA test for diagnosing CDI, in stool samples, was a limitation in our study; however, at the time of the study other tests/technologies were not available in Colombia (e.g. nucleic acid amplification tests/NAATs, glutamate dehydrogenase/GDH) [21]. In addition, the results observed could also be attributed to uncontrolled conditions in the pre-analytical phase; for instance, delays in the transport of some samples to the laboratory could result in toxin degradation by host enzymes [35,36]. Moreover, it would also be possible that some samples had very low level of toxin-producing *C*. *difficile* strains at the time of sampling [36].

Interestingly, we found two strains that were *tcd*A-/*tcd*B+ with the same profile of toxins. A possible explanation of these results may be that they can be attributed to different variants of the PaLoc. The most common variant type is *tcd*A-negative, *tcd*B-positive (*tcd*A-/*tcd*B+). Moreover, previous studies have reported deletions in the *tcd*A gene [37,38]; additionally, in the present study the NK2/NK3 primers did not amplify this gene in these variant-type strains. Furthermore, discordance between the negative cytotoxicity assay and the isolation of toxigenic ribotypes may be due to mixed *C*. *difficile* populations that could be present in the same patient's sample. According to this, toxin-negative colonies within a toxin-positive mixed population might have been selected for the toxicity assay.

There was no reduced susceptibility to vancomycin and metronidazole for any of the ribotypes analyzed. In contrast, in a previous study reported that ribotypes 027 and 001/072 as well as the ribotype 106 showed an elevated MIC to metronidazole [39]. Similarly, it was reported that ribotype 002 isolated from a Sweden patient who underwent long-term IV vancomycin therapy showed resistant to this antibiotic with MICs between 4–8 mg/L over a period of two years [40]. Moreover, the susceptibility to vancomycin and metronidazole observed in the isolates analyzed in the present study could reflect the low frequency of hypervirulent strains, at the time of sampling, which are more resistant to both antibiotics. The current guidelines for CDI management recommend both of these antibiotics as the first choice for treatment [34]. These results suggest to clinicians the advantage of adhering to the international guidelines in our setting, since these antibiotics are effective for the treatment of *C*. *difficile* infection.

Overall, the results obtained in the present study reinforce observations that MLVA is a useful technique that is able to differentiate strains, even in *C*. *difficile* isolates belonging to a same ribotype. Nonetheless, the variations observed, using this technique, in some isolates from a specific ribotype depend on which isolates are picked from the primary culture, as described elsewhere [28]. This dependence could affect epidemiological links among the strains analyzed, even though, an intra-hospital transmission of ribotypes 591 and 002 is presumed in hospitals B and A, respectively. No epidemiological association on time and space after analyzing the *C*. *difficile* clusters was detected, demonstrating the high molecular heterogeneity of strains circulating in Colombia. In addition, our results showed that patients infected by the dominant ribotypes 591, 106 and 002 have similar clinical characteristics.

## Conclusions

This study confirmed that MLVA is a useful discriminatory technique, and allowed us to identify a possible intra-hospital transmission of *C*. *difficile* ribotypes; this technique could be effective at tracing and tracking the source of contamination and is implementation could aid surveillance programs. Further research is needed to understand the clonal spread and long-term epidemiology of ribotypes 591, 106 and 002 as the predominant clones circulating in Colombia; in addition, a combination of two or more methods are desirable to provide essential information for investigating *C*. *difficile* population and clonal emergence.
